# Clinical characteristics of adenotonsillar hypertrophy in children: a retrospective analysis

**DOI:** 10.3389/fped.2026.1749566

**Published:** 2026-03-26

**Authors:** Liang Wu, Yingying Li, Wenbo Chen

**Affiliations:** Department of Otorhinolaryngology, Children’s Hospital of Nanjing Medical University, Nanjing City, Jiangsu, China

**Keywords:** adenoids, children, obstructive sleep apnea, risk factors, tonsil

## Abstract

**Objective:**

To explore the clinical characteristics of adenotonsillar hypertrophy (ATH) in children and determine its related clinical indicators.

**Methods:**

A retrospective cohort of 215 children with ATH who met predefined inclusion and exclusion criteria was identified from newly diagnosed patients presenting to the pediatric department of the study institution between March 1, 2024, and January 2025. Demographic characteristics, clinical data, and laboratory parameters were collected for subsequent univariate and multivariate analyses.

**Results:**

The cohort comprised 117 boys and 98 girls with a mean age of 5.81 ± 2.13 years, a mean tonsil-to-pharynx ratio (T/P ratio) of 0.60 ± 0.10, and a mean adenoid-to-nasopharynx ratio (A/N ratio) of 0.76 ± 0.08. Notably, A/N ratios differed significantly between children with and without snoring (0.78 ± 0.10 vs. 0.68 ± 0.04; *P* = 0.003), apnea (0.81 ± 0.10 vs. 0.75 ± 0.09; *P* < 0.001), and otitis media with effusion (0.81 ± 0.10 vs. 0.75 ± 0.10; *P* < 0.001). No significant differences in the T/P ratio were found across the assessed clinical symptoms (*P* > 0.05). Age, lowest oxygen saturation (LSaO_2_), and platelet distribution width demonstrated negative correlations with both the A/N ratio and T/P ratio. Conversely, snoring duration, the apnea–hypopnea index, and the platelet-to-lymphocyte ratio (PLR) showed positive correlations with both A/N and T/P ratios. Multivariate analysis of A/N ratios yielded a regression model, with *R*^2^ = 0.793 and *F* = 88.369 (*P* < 0.001). The multivariate regression model for the T/P ratio yielded *R*^2^ = 0.210 and *F* = 10.104 (*P* < 0.001).

**Conclusion:**

Adenoid and tonsillar hypertrophy have distinct associated factors, and age, PLR, and LSaO₂ aid in assessing pediatric ATH severity.

## 1 Introduction

Pediatric obstructive sleep apnea (OSA) is a sleep-related breathing disorder characterized by intermittent partial or complete upper airway obstruction during sleep, resulting in apneas and hypopneas. The condition leads to intermittent hypoxemia and frequent microarousals (1) and may increase the risk of cardiovascular, metabolic, and neurocognitive dysfunction as well as cancer-related mortality (2, 3). Obstructive sleep apnea is a multifactorial and multi-mechanistic disorder in which intermittent hypoxemia is recognized as the primary pathological mechanism. It may contribute to enhanced oxidative stress, sympathetic overactivity, systemic inflammation, metabolic dysregulation, and vascular inflammation with endothelial injury, potentially leading to multi-organ comorbidities in severe cases (4). Recent international studies indicate that the prevalence of pediatric OSA ranges from 1.2% to 5.7%. This condition may have irreversible long-term effects on neurocognitive function, behavioral development, growth, and cardiovascular health in children (5) and is thus gaining increasing attention from both society and parents. The pathogenesis of pediatric OSA involves multiple factors, including adenotonsillar hypertrophy (ATH), obesity, craniofacial abnormalities, and neuromuscular disorders. Among these, ATH is of particular concern, as it is considered one of the primary etiological factors contributing to pediatric OSA (6).

The adenoids and tonsils are vital immune organs—constituting key components of Waldeyer's ring—that play a crucial role in immune defense by capturing and eliminating pathogens entering the respiratory tract, activating local immune responses, and protecting the body from infection. The pathological essence of ATH involves the hyperplasia of lymphoid follicles, although its precise mechanisms remain incompletely understood. Current evidence suggests associations with recurrent infections (7), immune dysregulation (8), allergic factors (9), and hormonal influences (10). Adenoidectomy and/or tonsillectomy is currently recognized as the first-line treatment for ATH.

The current clinical assessment of ATH severity relies primarily on endoscopic examination and imaging studies. However, endoscopy is an invasive procedure often poorly tolerated by children, and imaging carries risks of radiation exposure and high costs. Consequently, identifying readily accessible and cost-effective indicators to assist in evaluating ATH severity is of significant clinical importance. With the increasing prevalence of ATH, research on this condition has intensified. Studies suggest that inflammatory markers may predict the severity of obstructive symptoms in pediatric OSA and the degree of systemic inflammatory response, highlighting their potential value for early disease assessment and comprehensive management. Nevertheless, the correlation between inflammatory markers and ATH severity remains inconclusive (11). This study therefore aims to investigate the association of demographic characteristics, clinical symptoms, medical history, and laboratory parameters with ATH severity and provide scientific evidence for the clinical evaluation and prognostic assessment of ATH in children.

## 2 Materials and methods

### 2.1 Study participants

A retrospective cohort of 215 children with ATH was identified from newly diagnosed patients presenting to the pediatric department of the study institution between March 1, 2024, and January 2025. The inclusion criteria were as follows: (1) patients with a concurrent diagnosis of adenoid hypertrophy (12) and tonsillar hypertrophy (13, 14); (2) children aged 2–14 years; (3) for children aged ≥8 years, the provision of informed consent and ability to comprehend and complete questionnaires; (4) for children aged <8 years, the provision of parental informed consent and the parent's ability to comprehend and complete questionnaires; and (5) patients with complete and reliable baseline medical records. The exclusion criteria were as follows: (1) patients with underlying medical conditions (e.g., craniofacial abnormalities; pulmonary, cardiac, or neurological disorders), (2) patients with severe skeletal malocclusion or substantial nasal obstruction, (3) patients with an adenoid-to-nasopharynx ratio (A/N ratio) > 0.88 or grade III tonsillar hypertrophy (i.e., obscuring essential anatomical landmarks for measurement), (4) children aged >12 years who had undergone a previous adenoidectomy or tonsillectomy for hypertrophy, and (5) the inability to cooperate with examination or treatment.

This study was approved by the Hospital Ethics Committee of the study hospital (approval no. 202403006-1).

Sample size was determined using G*Power 3.1 software (Heinrich Heine University Düsseldorf, Düsseldorf, Germany). Parameters for the primary outcome (the correlation between clinical/laboratory indicators and the A/N ratio) were set as follows: effect size (*r*) = 0.20, significance level (*α*) = 0.05 (two sided), and statistical power (1 – *β*) = 0.90. This yielded a minimum required sample size of 193 cases. To account for potential data loss in retrospective data collection, the sample size was expanded to 215 cases. A total of 247 eligible patients were initially screened, and 215 cases were ultimately enrolled after the inclusion and exclusion criteria were applied.

### 2.2 Study method

**Figure 1:**
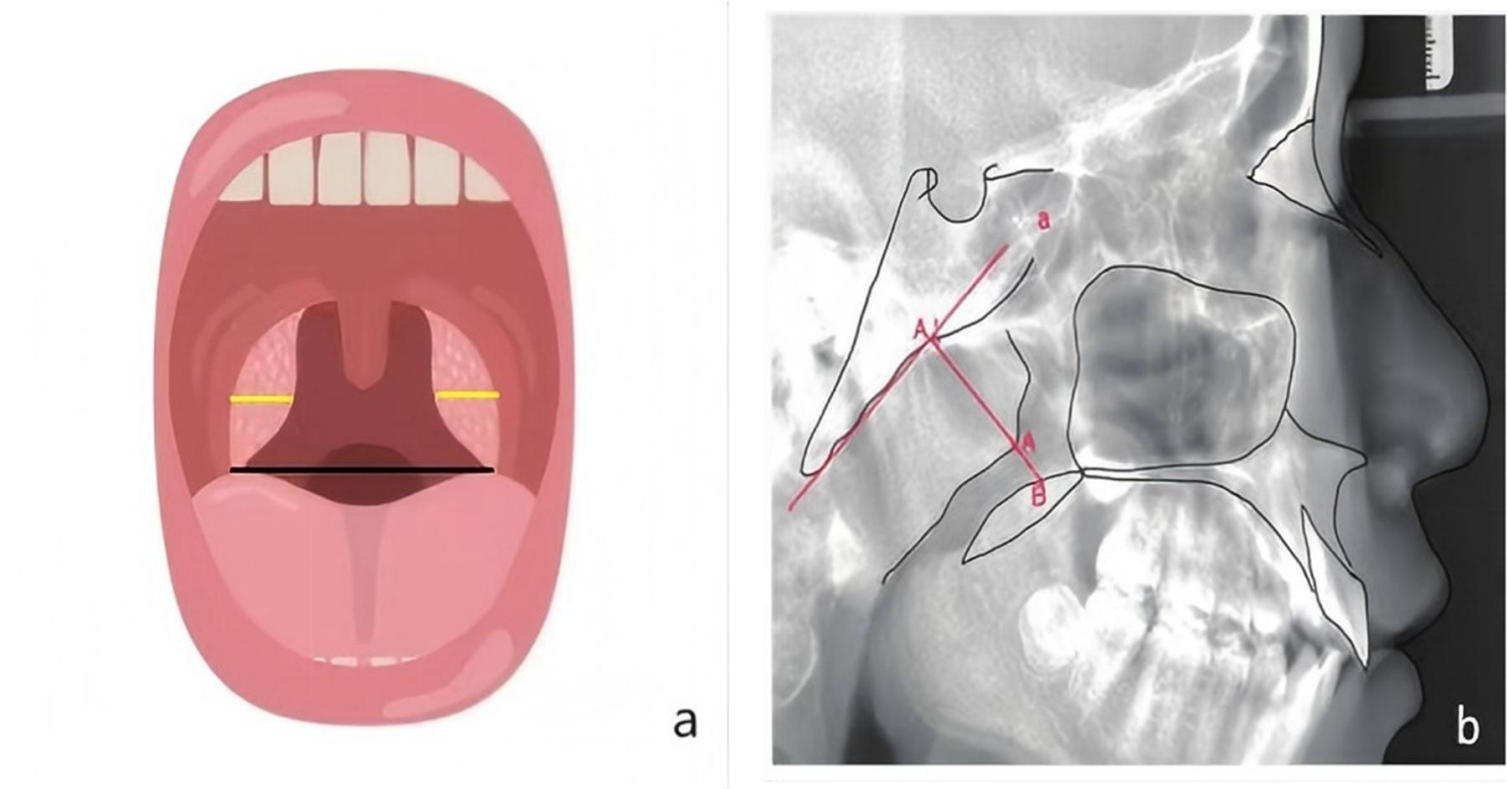
Measurement methods for the A/N ratio and T/P ratio. **(a)** The T/P ratio was used to evaluate the correlation between tonsil size and pharyngeal airway patency; the black line represents the pharyngeal airway width (defined as P). The sum of the two yellow lines represents the transverse width of the tonsil (defined as T). **(b)** Measurement method for the adenoid: A perpendicular line was drawn from the most convex point of the inferior margin of the adenoid (A) to the tangent line of the extracranial surface of the clivus **(a)**, with the foot of the perpendicular marked as A’. The reverse extension of this perpendicular line intersects the upper margin of the posterior end of the hard palate or the anterior-middle part of the soft palate at point B. The distance between A’ and B is defined as N, and the A/N ratio is calculated accordingly.

For the radiographic assessment of adenoid hypertrophy, lateral cephalometric radiography served as a critical diagnostic modality. Key radiographic features included thickening of the nasopharyngeal soft tissue and airway narrowing. The A/N ratio is a quantitative metric for evaluating adenoid hypertrophy and was defined and measured in this study as follows (12): for anatomical landmarks, (a) point A was defined as the most prominent point of the adenoid tissue shadow on the lateral cephalogram, (b) point N was defined as the posterior margin of the hard palate [or the anterior margin of the basiocciput (12)], and (c) point P was defined as the posterior wall of the nasopharynx at the level of point N; (d) adenoid thickness was defined as the vertical distance from point A to the posterior pharyngeal wall, and (e) nasopharynx width was defined as the distance between the anterior margin of the nasopharyngeal airway (at point N) and the posterior pharyngeal wall (point P). The formula used to calculate the A/N ratio was as follows: adenoid thickness (A)/nasopharynx width (N). According to the diagnostic criteria established by the *Chinese Journal of Radiology* (12), A/N ratios are classified into three grades: mild hypertrophy (0.50–0.60), moderate hypertrophy (0.61–0.75), and severe hypertrophy (>0.75–0.88). This grading system provides essential guidance for clinical diagnosis and therapeutic decision-making. All radiographs were independently evaluated by two otolaryngologists with ≥10 years of clinical experience. Discrepancies were resolved by consensus with a third equally qualified otolaryngologist. Patients meeting both the inclusion and exclusion criteria were enrolled (Figure 1a).

Tonsil examination was performed with the patient seated and the head slightly extended. Patients were instructed to open their mouths fully and phonate/ɑː/ while an examiner depressed the anterior two-thirds of the tongue with a tongue depressor. Under adequate illumination, tonsillar size was classified as follows: Grade I – tonsils medial to the palatopharyngeal arch, Grade II – tonsils extending beyond the palatopharyngeal arch but not reaching the midline of the posterior pharyngeal wall, or Grade III – tonsils extending to or beyond the pharyngeal midline. Tonsil-to-pharynx (T/P) ratios were measured following Shintani's methodology (13), with the following specific definitions of anatomical landmarks: (a) tonsil width was defined as the maximum horizontal distance from the medial margin of one palatine tonsil to the medial margin of the contralateral tonsil, and (b) pharynx width was defined as the horizontal distance between the two palatopharyngeal arches at the same level as the tonsil width measurement (avoiding compression of the pharyngeal mucosa during measurement). The formula used to calculate the T/P ratio was as follows: tonsil width (T)/pharynx width (P). Higher T/P ratios indicated greater severity, with values exceeding 0.5 indicating potential tonsillar hypertrophy (14). Inter-observer agreement for T/P ratio measurement was represented by an intraclass correlation coefficient of 0.89 (95% confidence interval: 0.84–0.93), confirming reliable consistency (Figure 1b).

The selection of assessment methods was tailored to the anatomical characteristics of adenoids and tonsils. Adenoids are concealed in the nasopharynx, so lateral cephalometric radiography was used for the non-invasive quantitative evaluation of the A/N ratio [validated by (12, 14)]. Tonsils are accessible in the oropharynx, so a clinical examination with a T/P ratio measurement [per (13)] was adopted for direct, radiation-free assessment. Strict quality control (a double-blinded radiograph review and standardized clinical procedures) minimized measurement bias. Both metrics are widely recognized in pediatric ATH research (14), ensuring comparability in severity evaluation.

### 2.3 Data collection

Data on patients’ demographic characteristics, clinical parameters, and laboratory indices were collected. Demographic characteristics included gender, age, habitual snoring duration, and body mass index. Clinical parameters included snoring, open-mouth breathing, apnea, disturbed sleep, nasal congestion, rhinorrhea, aural fullness, hearing loss, dysphagia, slurred speech, adenoid facies, the T/P ratio, the apnea–hypopnea index (AHI), lowest oxygen saturation (LSaO_2_), the A/N ratio, and the presence of secretory otitis media (SOM).

Laboratory indices comprised complete blood count (CBC)-derived parameters, including the neutrophil-to-lymphocyte ratio, platelet-to-lymphocyte ratio (PLR), monocyte-to-lymphocyte ratio, mean platelet volume, and platelet distribution width (PDW). Additional laboratory indices included albumin, the albumin-to-globulin ratio, fibrinogen, fibrinogen degradation products, and D-dimer. To collect samples for CBC testing, peripheral venous blood was collected concurrently with lateral cephalometric radiography (within 24 h) at the time of the initial ATH diagnosis. All patients were in a fasting state (≥8 h) during blood collection, and prior to sampling, patients were screened to exclude acute infections (e.g., fever, cough, sore throat), a history of respiratory infections or anti-infective treatment within 1 month, and other acute conditions (e.g., allergic episodes, diarrhea) that might interfere with the CBC results. This ensured that the CBC-derived indices used reflected the stable physiological state of children with ATH, minimizing the confounding effects of external factors.

### 2.4 Statistical analysis

Statistical analyses were performed using SPSS software (version 26.0). The normality of continuous variables was assessed using the Kolmogorov–Smirnov test. Normally distributed data were presented as mean ± standard deviation and compared using independent sample *t*-tests. Pearson correlation analysis was employed to evaluate associations, multivariate linear regression was utilized for multifactor analysis, and the influencing factors of the A/N and T/P ratios in patients were further explored. With the A/N and T/P ratios as dependent variables and variables with statistically significant differences in univariate analysis as independent variables, stepwise regression analysis was performed. The stepwise regression method was used to include and exclude independent variables (*α*_entry_ = 0.05, *α*_removal_ = 0.1), and influencing factors with interaction effects were eliminated. A two-sided *P*-value <0.05 was considered statistically significant.

## 3 Results

### 3.1 Clinical characteristics

Among the 215 patients in the cohort, there were 117 boys and 98 girls, with a mean age of 5.81 ± 2.13 years, a mean T/P ratio of 0.60 ± 0.10, and a mean A/N ratio of 0.76 ± 0.08. The top three clinical symptoms were snoring (207 cases, 96.3%), nasal obstruction (190 cases, 88.4%), and adenoid facies (153 cases, 71.2%). The distribution of other clinical characteristics is detailed in Table 1.

**Table 1:** Clinical characteristics.

Item	Number or x ± s	Percentage（%）
Gender		
Male	117	54.4
Female	98	45.6
Snoring	207	96.3
Open-mouth breathing	109	50.7
Apnea	91	42.3
Sleep disturbance	150	69.8
Nasal obstruction	190	88.4
Rhinorrhea	50	23.3
Aural fullness	10	4.7
Hearing loss	2	0.9
Dysphagia	40	18.6
Dysarthria	65	30.2
Adenoid face	153	71.2
Secretory otitis media	96	44.7
Age (year)	5.81 ± 2.13	-
Snoring duration (months)	14.46 ± 17.66	-
BMI (kg/m^2^)	18.53 ± 1.43	-
T/*P* value	0.60 ± 0.10	-
AHI	18.86 ± 13.26	-
LSaO₂(%)	91.34 ± 82.61	-
A/N value	0.76 ± 0.08	-
NLR	2.52 ± 1.18	-
PLR	155.55 ± 99.52	-
MLR	0.23 ± 0.15	-
MPV (fL)	13.21 ± 10.32	-
PDW (%)	12.49 ± 11.56	-
ALB (g/L)	51.27 ± 44.05	-
AGR	2.53 ± 1.86	-
FIB (g/L)	3.64 ± 2.40	-
FDP (mg/L)	4.21 ± 2.54	-
D-D (*μ*g/mL)	0.88 ± 0.40	-

BMI, body mass index; AHI, apnea–hypopnea index; LSaO2, lowest oxygen saturation; NLR, neutrophil-to-lymphocyte ratio; PLR, platelet-to-lymphocyte ratio; MLR, monocyte-to-lymphocyte ratio; MPV, mean platelet volume; PDW, platelet distribution width; ALB, additional laboratory indices including albumin; AGR, albumin-to-globulin ratio; FIB, fibrinogen; FDP, fibrinogen degradation products; DD, D-dimer.

### 3.2 Correlation analysis between categorical data and adenoid-to-nasopharynx and tonsil-to-pharynx ratios

There were statistically significant differences in the A/N ratio between patients with and without snoring (0.78 ± 0.10 vs. 0.68 ± 0.04, *P* = 0.003), with and without apnea (0.81 ± 0.10 vs. 0.75 ± 0.09, *P* < 0.001), and with and without SOM (0.81 ± 0.10 vs. 0.75 ± 0.10, *P* < 0.001) (all *P* < 0.05). There were no statistically significant differences in the T/P ratio among the various clinical symptoms (*P* > 0.05), as shown in Table 2.

**Table 2:** Correlation analysis between categorical data and degree of adenoidal hypertrophy (A/N ratio) and degree of tonsillar hypertrophy (T/P ratio).

Variable	A/N value		T/P value	
	x ± s	*P* value	x ± s	*P* value
Gender		0.127		0.902
Male (*n* = 117)	0.77 ± 0.10		0.60 ± 0.03	
Female (*n* = 98)	0.79 ± 0.10		0.60 ± 0.03	
Snoring		0.003		0.384
With (*n* = 207)	0.78 ± 0.10		0.60 ± 0.03	
Without (*n* = 8)	0.68 ± 0.04		0.61 ± 0.02	
Open-mouth breathing		0.248		0.856
With (*n* = 109)	0.79 ± 0.11		0.60 ± 0.03	
Without (*n* = 106)	0.77 ± 0.10		0.60 ± 0.03	
Apnea		<0.001		0.370
With (*n* = 91)	0.81 ± 0.10		0.60 ± 0.03	
Without（*n* = 124）	0.75 ± 0.09		0.60 ± 0.03	
Sleep disturbance		0.271		0.393
With (*n* = 150)	0.78 ± 0.11		0.60 ± 0.03	
Without (*n* = 65)	0.77 ± 0.09		0.59 ± 0.03	
Nasal obstruction		0.421		0.594
With (*n* = 190)	0.78 ± 0.10		0.59 ± 0.02	
Without (*n* = 25)	0.76 ± 0.11		0.60 ± 0.03	
Rhinorrhea		0.229		0.525
With（*n* = 50）	0.79 ± 0.09		0.59 ± 0.03	
Without (*n* = 165)	0.77 ± 0.10		0.60 ± 0.03	
Aural fullness		0.405		0.697
With (*n* = 10)	0.75 ± 0.10		0.59 ± 0.03	
Without (*n* = 205)	0.78 ± 0.10		0.60 ± 0.03	
Hearing loss		0.694		0.902
With (*n* = 2)	0.75 ± 0.20		0.60 ± 0.03	
Without (*n* = 213)	0.78 ± 0.10		0.60 ± 0.03	
Dysphagia		0.960		0.902
With (*n* = 40)	0.78 ± 0.09		0.60 ± 0.03	
Without (*n* = 175)	0.78 ± 0.11		0.60 ± 0.03	
Dysarthria		0.489		0.339
With (*n* = 65)	0.77 ± 0.09		0.59 ± 0.03	
Without (*n* = 150)	0.78 ± 0.11		0.60 ± 0.03	
Adenoid face		0.107		0.366
With (*n* = 153)	0.79 ± 0.10		0.60 ± 0.03	
Without (*n* = 62)	0.76 ± 0.11		0.59 ± 0.03	
Secretory otitis media		<0.001		0.274
With (*n* = 96)	0.81 ± 0.10		0.60 ± 0.30	
Without (*n* = 119)	0.75 ± 0.10		0.60 ± 0.30	

### 3.3 Correlation analysis between quantitative data and adenoid-to-nasopharynx and tonsil-to-pharynx ratios

Consistently, age, LSaO_2_, and PDW were negatively correlated with the A/N ratio, whereas snoring duration, the AHI, and the PLR were positively correlated with the A/N ratio. The results of T/P ratio correlation analysis were consistent with those of A/N ratio analysis, as shown in Table 3.

**Table 3:** Correlation between quantitative data and degree of adenoidal hypertrophy (A/N value) and degree of tonsillar hypertrophy (T/*P* value).

Variable	A/N value		T/*P* value	
	*r* value	*P* value	*r* value	*P* value
Age (year)	−0.250	<0.001	−0.174	0.011
Snoring duration	0.832	<0.001	0.265	<0.001
BMI (kg/m^2^)	0.104	0.128	0.111	0.104
AHI (%)	0.782	<0.001	0.153	0.025
LSaO₂ (%)	−0.283	<0.001	−0.375	<0.001
NLR	−0.016	0.817	0.018	0.794
PLR	0.354	<0.001	0.179	0.009
MLR	0.020	0.767	0.052	0.447
MPV (fL)	−0.010	0.888	−0.133	0.052
PDW (%)	−0.301	<0.001	−0.324	<0.001
ALB (g/L)	0.008	0.903	−0.047	0.498
AGR	−0.057	0.402	−0.032	0.645
FIB (g/L)	−0.065	0.346	0.017	0.801
FDP (mg/L)	0.101	0.140	0.097	0.155
D-D (μg/mL)	0.053	0.440	0.125	0.068

BMI, body mass index; AHI, apnea–hypopnea index; LSaO2, lowest oxygen saturation; NLR, neutrophil-to-lymphocyte ratio; PLR, platelet-to-lymphocyte ratio; MLR, monocyte-to-lymphocyte ratio; MPV, mean platelet volume; PDW, platelet distribution width; ALB, additional laboratory indices including albumin; AGR, albumin-to-globulin ratio; FIB, fibrinogen; FDP, fibrinogen degradation products; DD, D-dimer.

### 3.4 Multiple linear regression

The A/N ratio, apnea, and SOM were excluded during the process of variable inclusion and exclusion. The obtained regression model had an *R*^2^ value of 0.793, indicating that the remaining variables could explain 79.3% of the variation in the A/N ratio, with *F* = 88.369 and *P* < 0.001; the Durbin–Watson index was 1.195, suggesting that there was no correlation among independent variables in the model; the significance test results of the remaining independent variables in the model all showed *P* < 0.05, proving that the independent variables were statistically significant in the model and should be retained; and the variance inflation factor (VIF) values of the independent variables were all far less than 10, so there was no collinearity among the independent variables. According to the partial regression coefficients in the model, the order of influence intensity of independent variables on the A/N ratio was as follows: snoring duration > AHI > age > PDW > PLR > snoring > LSaO_2_ (Table 4).

**Table 4:** Multiple linear regression of A/N value.

Item	B	SE	Standardized B	*P* value	95%CI lower limit	95%CI upper limit	VIF
Snoring	0.038	0.016	0.079	0.017	0.007	0.069	1.071
Apnea	0.009	0.006	0.048	0.153	−0.003	0.021	1.121
Secretory otitis media	0.012	0.006	0.064	0.057	0.000	0.024	1.110
Age	−0.008	0.001	−0.177	0.000	−0.010	−0.005	1.071
Snoring duration	0.003	0.000	0.631	0.000	0.003	0.004	2.928
AHI	0.011	0.002	0.288	0.000	0.007	0.015	2.366
LSaO₂	−0.002	0.001	−0.094	0.016	−0.004	0.000	1.488
PLR	−0.001	0.000	−0.113	0.001	−0.001	0.000	1.172
PDW	0.046	0.014	0.137	0.002	0.018	0.074	1.801
Constant	0.302	0.151	-	0.047	0.003	0.601	-

R = 0.896, R^2^ = 0.802, adjusted R^2^ = 0.793, F = 88.369, *P* < 0.05. AHI, apnea–hypopnea index; LSaO2, lowest oxygen saturation; PLR, platelet-to-lymphocyte ratio; PDW, platelet distribution width.

In terms of the T/P ratio, snoring duration, the AHI, and PDW were excluded during the process of variable inclusion and exclusion. The obtained regression model had an *R*^2^ value of 0.210, indicating that the remaining variables could explain 21.0% of the variation in the T/P ratio, with *F* = 10.104 and *P* < 0.001; the Durbin–Watson index was 1.621, suggesting that there was no correlation among independent variables in the model; the significance test results of the remaining independent variables in the model all showed *P* < 0.05, proving that the independent variables were statistically significant in the model and should be retained; and the VIF values of the independent variables were all far less than 10, so there was no collinearity among the independent variables. According to the partial regression coefficients in the model, the order of influence intensity of independent variables on the T/P ratio was as follows: LSaO_2_ > PLR > age, as shown in Table 5.

**Table 5:** Multiple linear regression of T/*P* value.

Item	B	SE	Standardized B	*P* value	95%CI lower limit	95%CI upper limit	VIF
Age	−0.002	0.001	−0.137	0.030	−0.004	0.001	1.029
Snoring duration	0.001	0.001	0.041	0.696	0.001	0.001	2.838
AHI(%)	0.001	0.001	−0.020	0.830	−0.003	0.002	2.325
LSaO₂(%)	−0.002	0.001	−0.282	0.000	−0.004	−0.001	1.436
PLR	0.001	0.001	0.144	0.032	0.001	0.001	1.165
PDW	−0.016	0.009	−0.152	0.068	−0.033	0.001	1.796
(Constant)	0.965	0.092	-	0.000	0.783	1.147	-

*R* = 0.483, *R^2^* = 0.233, adjusted *R^2^* = 0.210, *F* = 10.104, *P* < 0.05. AHI, apnea–hypopnea index; LSaO2, lowest oxygen saturation; PLR, platelet-to-lymphocyte ratio; PDW, platelet distribution width.

## 4 Discussion

Based on multivariate linear regression analysis, the independent factors correlating with the A/N ratio were ranked from high to low according to their intensity of influence as follows: snoring duration > AHI > age > PDW > PLR > snoring > LSaO_2_. This ranking reflects the relative contribution of each factor to adenoid hypertrophy severity, providing targeted insights for clinical assessment. These findings align with previous research on pediatric ATH and further clarify the hierarchical importance of clinical and laboratory factors in regulating adenoid hypertrophy severity. Below, we discuss key associations and clinical implications in detail.

Snoring duration emerged as the most influential factor; this may be attributed to the long-term mechanical stimulation of the nasopharyngeal mucosa by airflow vibration during snoring. This persistent stimulation could promote lymphoid follicle hyperplasia in adenoid tissue (15), thereby exacerbating hypertrophy. The AHI, the second most impactful factor, is a core indicator of sleep-disordered breathing frequency and severity. An elevated AHI suggests more frequent obstructive events, which may induce chronic intermittent hypoxemia and systemic inflammatory responses (6, 16), further aggravating adenoid hypertrophy.

Age, the third key factor, exhibited a negative correlation with the A/N ratio, indicating that adenoid hypertrophy is more severe in younger children. This aligns with the physiological characteristic of adenoid tissue, which undergoes gradual regression with age as the immune system matures (17), reducing a patient's susceptibility to hypertrophy-inducing factors such as recurrent infections and immune dysregulation.

Among the laboratory indices, PDW and PLR were successive influential factors. A potential mechanism for PDW's association is that severe ATH may increase OSA-related intermittent hypoxemia risk (6), which modulates platelet activation and size variability via compensatory physiological responses in children (16). Specifically, hypoxemia-induced oxidative stress and systemic inflammation can trigger a compensatory hypercoagulable state in pediatric OSA, characterized by abnormal PDW, as confirmed by Wang et al. (16), who linked abnormal PDW in children with sleep-disordered breathing to hypoxic stress-induced compensatory platelet activation. The PLR, a novel inflammatory marker (18), was positively correlated with the A/N ratio. This was consistent with persistent ATH-induced inflammation, which promotes platelet proliferation and lymphopenia, leading to elevated PLRs. Notably, relevant studies on pediatric OSA and ATH have supported this association. Yuan et al. (11) reported that the PLR was significantly higher in children with moderate-to-severe OSA than in mild cases, and Park et al. (8) found a positive correlation between the PLR and tonsillar hypertrophy in children with obesity and ATH. Our findings further validate the PLR as a non-invasive inflammatory indicator for assessing ATH severity in pediatric patients.

Snoring and LSaO_2_ had relatively weaker influences on the A/N ratio. Snoring, as an early clinical manifestation of upper airway obstruction, may reflect initial adenoid enlargement but is less impactful than long-term snoring duration, and LSaO_2_, a downstream indicator of hypoxemia, reflects the physiological consequence of ATH-induced airway obstruction rather than a direct driver of adenoid hypertrophy (15).

Notably, although ATH is a key etiological factor for pediatric OSA (6), the A/N ratio does not directly equate to OSA severity, as OSA is multifactorial. Additionally, children with SOM were found to have higher A/N ratios in univariate analysis, indicating a potential association with adenoid hypertrophy severity. This may be related to adenoid-induced Eustachian tube dysfunction (18), although SOM was excluded from the final regression model due to the statistical criteria.

Consistent with the ranking described above, age emerged as a critical factor negatively correlating with the A/N ratio, indicating that the degree of adenoidal hypertrophy gradually decreases with increasing age. This may be associated with the physiological regression of adenoid tissue over time. Numerous studies worldwide have reported that adenoidal hypertrophy is linked to recurrent infections, immune dysregulation, and allergic reactions (17). As children grow older, their immune systems mature progressively, culminating in a reduced risk of adenoidal hypertrophy-inducing factors such as infections and allergies. Thus, the degree of adenoidal hypertrophy may diminish with age. It is also important to note that although ATH is a key etiological factor for pediatric OSA (6), its severity does not directly equate to OSA severity, as OSA is a multifactorial disorder influenced by other factors, including obesity and craniofacial abnormalities.

Beyond the factors included in the regression model, univariate analysis identified a potential association between SOM and adenoid hypertrophy severity that was characterized by effusion in the middle ear, an intact tympanic membrane, and hearing loss (also known as otitis media with effusion) (19). Otitis media is the most common childhood ear disease and cause of hearing loss in children; however, its symptoms are often inconspicuous and tend to be overlooked in the early stage. Early hearing loss in children may lead to irreversible lifelong sequelae, affecting children's language development and social relationships (20). The etiology of SOM is multifactorial, and among these factors, adenoidal hypertrophy plays an important role in the pathogenesis of SOM in children (21). The results of this study indicate that children with SOM may have a more severe degree of adenoidal hypertrophy.

This study has certain limitations. First, its single-center retrospective design implies a singularity of data sources, introducing a risk of bias. Second, although the AHI was used as the primary polysomnographic index to assess sleep ventilation function, the obstructive AHI (OAHI), which excludes central events and better reflects obstructive pathology, was not separately analyzed or reported. This is because OAHI data were not fully available in partial routine polysomnographic reports from the retrospective cohort, limiting the specificity of sleep-related outcome assessments. Finally, the multivariate regression model for the T/P ratio exhibits relatively low explanatory power, indicating that a substantial proportion of the variability in tonsillar hypertrophy remains unexplained. In future studies, we will adopt multicenter designs; expand the scope of cases (e.g., including more children with varying degrees of ATH and healthy children); optimize data collection and analysis methods (by including incorporating additional potential confounding variables, such as genetic factors, environmental exposures, and craniofacial measurements; using more precise imaging modalities to assess tonsillar volume; and prioritizing OAHI as the core polysomnographic indicator to enhance the specificity of obstructive sleep event assessment); and make greater use of prospective studies to reduce the impact of recall bias and other confounding factors on the research results, improve the explanatory power of statistical models, and provide more robust evidence for clinical practice.

## 5 Conclusion

In summary, factors correlating with the severity of adenoid hypertrophy and tonsillar hypertrophy are distinct and should be separated. Younger age, longer snoring duration, a higher AHI, a higher PLR, lower PDW, and lower LSaO_2_ are associated with adenoid hypertrophy severity, whereas, for tonsillar hypertrophy, younger age, a higher PLR, and lower LSaO_2_ are the key correlating factors. These findings provide a targeted theoretical basis for the clinical diagnosis and severity assessment of pediatric ATH.

## Data Availability

The original contributions presented in the study are included in the article/Supplementary Material, further inquiries can be directed to the corresponding author.
